# SSR-Based Genetic Diversity, Population Structure, and Marker–Trait Associations for Popping-Related Traits in Popcorn Germplasm

**DOI:** 10.3390/genes17060690

**Published:** 2026-06-12

**Authors:** Lin Yang, Jialin Yu, Ning Wang, Huilin Yu, Dan You, Yanxing Wang, Shuai Shao, Xin Qi, Yang Zhang, Yuqun Wu

**Affiliations:** Maize Research Institute, Liaoning Academy of Agricultural Sciences, Shenyang 110161, China; younglinlin1993@126.com (L.Y.); yjl890803@sina.com (J.Y.); ningwang1993@126.com (N.W.); yuhuilin19831222@163.com (H.Y.); danyou6811@126.com (D.Y.); yanxing1964@126.com (Y.W.); shaoshuaispring@126.com (S.S.); lnsnkyqx@163.com (X.Q.)

**Keywords:** popcorn, SSR markers, genetic diversity, population structure, marker–trait association

## Abstract

**Background/Objectives**: Popcorn (*Zea mays* L. var. *everta*) is an important specialty maize type; however, the genetic variation underlying popping-related quality traits remains insufficiently characterized in breeding. **Methods**: In this study, 18 popcorn inbred lines were analyzed using 25 simple sequence repeat (SSR) markers distributed across all 10 maize chromosomes, and 16 lines were further evaluated for popping performance and image-based flake morphology. **Results**: Substantial phenotypic variation was observed among the tested lines, with expansion volume ranging from 173.33 to 343.33 mL and expandability ranging from 16.79- to 32.46-fold. Image-based analysis of 957 popped kernels revealed continuous variation in flake circularity, indicating that flake morphology represents a quantitative trait rather than a strictly discrete classification. SSR analysis detected 2 to 11 alleles per locus, with polymorphism information content values ranging from 0.05 to 0.85, indicating moderate-to-high genetic diversity among the tested lines. Principal component analysis (PCA), unweighted pair group method with arithmetic mean (UPGMA) clustering, and population structure analysis revealed clear genetic differentiation and heterogeneous genetic backgrounds within the germplasm collection. Marker–trait association analysis identified several putative SSR loci associated with expansion efficiency, flake morphology, pericarp retention, and popping dynamics. Notably, marker M18 was putatively associated with both expansion volume and expandability. **Conclusions**: Based on these results, a conceptual framework was proposed in which popping-related traits were organized into partially independent but interconnected functional modules. Overall, this study provides SSR-based genetic information for popcorn germplasm characterization and offers preliminary marker resources for quality-oriented popcorn breeding.

## 1. Introduction

Popcorn (*Zea mays* L. var. *everta*) is a specialty maize type valued for its popping ability and sensory quality, which largely determine its commercial value and consumer acceptance [1]. Popping performance is typically evaluated by expansion volume, flake morphology, including mushroom and butterfly types, and texture, with expansion volume widely regarded as the most important industrial trait [2]. Over the past three decades, popcorn breeding in China has progressed from the breeding of synthetic varieties and officially released single-cross hybrids at both the provincial and national levels through to the recent quality-oriented breeding targeting specialized flake types [3]. In China, evaluations of popping quality and the requirements for raw kernels are guided by the agricultural industry standard NY/T 523−2020 [4], which also provides a reference framework for processed popcorn product standards. Comparative assessments of commercial popcorn products in China have indicated substantial variation in popping performance among brands and product types, underscoring the need for more consistent quality evaluation and improvement [5].

Popping quality is highly sensitive to postharvest and processing conditions [6]. In a representative mushroom-type variety, kernel moisture exhibited a unimodal relationship with expansion, with an optimal range of 13.4–13.8%, whereas increased maturity was positively associated with both expansion and the mushroom-flake rate [7]. More broadly, popping is a complex physical and biological process governed by the interaction between internal pressure generated during heating and the structural integrity of the kernel [1,8]. In particular, the structural features of the pericarp and endosperm, including pericarp thickness, vitreousness, and the organization of the starch–protein matrix, play critical roles in determining rupture behavior and flake formation [9,10]. Previous studies have shown that smaller and harder kernels with higher proportions of vitreous endosperm tend to exhibit higher expansion volume and fewer unpopped kernels [11]. Early studies on popcorn mainly evaluated phenotypes and the physical mechanisms underlying popping behavior, particularly the effects of moisture content, heating conditions, and kernel hardness on expansion volume and flake quality [1,7,12]. Together, these findings highlight that standardized quality evaluation, appropriate postharvest management, and a better understanding of kernel structural traits are all essential for the improvement of popcorn popping quality.

From a breeding perspective, grain yield and popping expansion are the two major target traits in popcorn improvement, because they jointly determine agronomic performance and commercial value [13]. However, these traits are not always easily optimized simultaneously, and commercial popcorn germplasm with high popping expansion may perform poorly for grain yield outside its target adaptation region [14]. In addition, environmental factors such as water limitation can substantially reduce both grain yield and expanded popcorn volume [15], indicating that popcorn quality and productivity are jointly shaped by genotype, environment, and their interactions. Therefore, improving popcorn requires not only the phenotypic evaluation of popping-related traits, but also genetic characterization that can help breeders identify useful parental materials and better understand the biological basis of trait variation.

Integrating molecular marker information with multi-trait phenotyping may accelerate the development of high-quality popcorn germplasms [16]. In recent years, genomics-assisted approaches, including QTL mapping, GWAS, and meta-QTL analysis, have begun to reveal the genetic basis of popping-related traits and kernel quality attributes in popcorn and maize, highlighting their complex and polygenic architecture [17,18,19,20]. Population-level analyses have also revealed the clear genetic structure and reduced diversity in some modern breeding groups, suggesting potential constraints for future improvement [19]. However, compared with quality traits in other types of maize, the genetic basis of popping-related traits in popcorn remains insufficiently explored and has not yet been effectively translated into practical breeding applications. In particular, genetic information related to flake morphology and pericarp-related traits remains limited.

SSR markers have been widely used in maize genetic studies because of their high polymorphism, reproducibility, and cost-effectiveness, making them particularly suitable for evaluating genetic diversity and population structure in breeding materials [21,22,23]. Previous studies have shown that SSR markers are effective for assessing genetic diversity and population structure in popcorn germplasm, thereby providing useful information for parent selection and germplasm utilization in breeding programs [24,25]. In addition, popping-related traits are quantitatively inherited and typically influenced by multiple loci as well as environmental factors [26], which underscores the importance of integrating molecular marker analysis with phenotypic evaluation in popcorn improvement. In parallel, recent advances in image-based phenotyping have led to the establishment of efficient and objective approaches for the quantitative characterization of complex morphological traits that are difficult to assess using conventional methods [27]. By enabling high-throughput, non-destructive and reproducible measurements, image analysis techniques have been increasingly applied in crop research, particularly for grain shape, size and structural features [28,29]. Such methods allow for morphological traits, including popcorn flake shape parameters, to be treated as quantitative variables, thereby facilitating their integration into genetic and statistical analyses. Therefore, combining SSR markers with high-resolution phenotyping provides a practical framework for characterizing popcorn quality traits and generating preliminary marker information to support quality-oriented breeding.

The objectives of this study were to: (i) characterize the genetic diversity and population structure of popcorn inbred lines using SSR markers; (ii) evaluate phenotypic variation in popping-related traits and image-based flake morphology; (iii) explore putative marker–trait associations related to expansion efficiency, flake morphology, pericarp retention, and popping dynamics; and (iv) propose an integrated framework linking SSR loci with major components of popping performance. These results are expected to provide molecular information for popcorn germplasm characterization and preliminary marker resources for quality-oriented popcorn improvement.

## 2. Results

### 2.1. Evaluation of Uniformity in Popcorn Inbred Lines

Uniformity was first evaluated in the initial set of 20 popcorn inbred lines. Several lines, including POP14 and POP16, exhibited relatively high overall uniformity scores (Figure S1A). Variation was observed across individual uniformity components, including plant height, ear position, tassel morphology, and flowering synchrony, with different lines showing advantages for different traits (Figure S1B).

After field planting, two lines, POP18 and POP20, failed to germinate and were excluded from further evaluation. Among the remaining 18 lines, two lines, POP16 and POP17, were not included in the popping test due to insufficient seed harvest, although they were retained for SSR analysis (Table S1). These results reflect both the diverse origins of the conserved germplasm and the practical challenges associated with maintaining line uniformity and seed availability during long-term preservation and regeneration.

### 2.2. Popping-Related Traits

The overall popping-related phenotypic performance of the 16 popcorn inbred lines is presented in Table 1; the raw phenotypic data for all measured popping-related traits are provided in Tables S2 and S3. Considerable phenotypic variation was observed among lines for all evaluated traits. The expansion volume (EV) ranged from 173.33 to 343.33 mL, whereas expandability ranged from 16.79- to 32.46-fold across the evaluated lines. The first pop time also exhibited noticeable variation, spanning from 31.00 to 51.67 s. Differences were further detected in unpopped ratio (UR), pericarp retention (PR), and flake-type distribution. Notably, a high proportion of mushroom-shaped flakes (40%) was observed in one inbred line, whereas most lines predominantly produced butterfly-type flakes. Overall, these results indicate substantial phenotypic diversity within the studied germplasm.

### 2.3. Image-Based Flake Shape Classification

**Figure 1:**
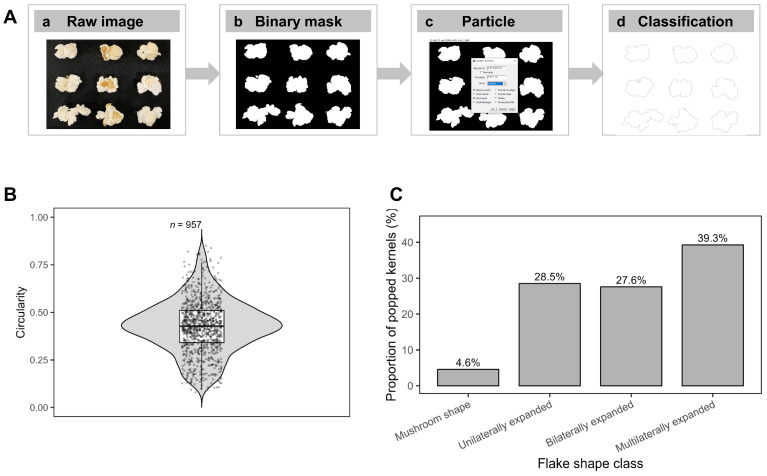
Image-based phenotyping classification of popped popcorn kernels. (**A**) Flowchart of the image-based phenotyping, including raw image acquisition, binary mask generation, particle segmentation, and shape classification. (**B**) Distribution of circularity values for all analyzed popped kernels (*n* = 957). (**C**) Proportion of popped kernels assigned to each flake shape class based on circularity thresholds: mushroom-shaped flakes, 0.67–1.00; unilaterally expanded butterfly flakes, 0.48–0.67; multilaterally expanded butterfly flakes, 0.40–0.48; and bilaterally expanded butterfly flakes, 0.32–0.40.

The workflow of the image-based phenotyping system is illustrated in Figure 1A, including raw image acquisition, binary mask generation, particle segmentation, and automated shape classification. A total of 957 popped kernels were analyzed across the tested inbred lines, providing a dataset for morphological evaluation. The distribution of circularity values (Figure 1B) showed a continuous pattern, with most kernels ranging between approximately 0.25 and 0.50. The unimodal yet moderately skewed distribution indicated substantial variation in flake morphology among the analyzed samples rather than discrete clustering into distinct shape types. Based on predefined circularity thresholds, popped kernels were categorized into four flake shape classes (Figure 1C). Multilaterally expanded flakes accounted for the largest proportion (39.3%), followed by unilaterally expanded (28.5%) and bilaterally expanded (27.6%) flakes. Mushroom-shaped flakes represented only 4.6% of the total analyzed kernels. The predominance of butterfly-type flakes suggests that most tested materials tend to produce irregular expansion patterns rather than the compact morphology characteristic of mushroom-type flakes.

This image-based classification provided quantitative flake shape variables that were used in the subsequent exploratory marker–trait association analysis.

### 2.4. SSR Marker Analysis

**Figure 2:**
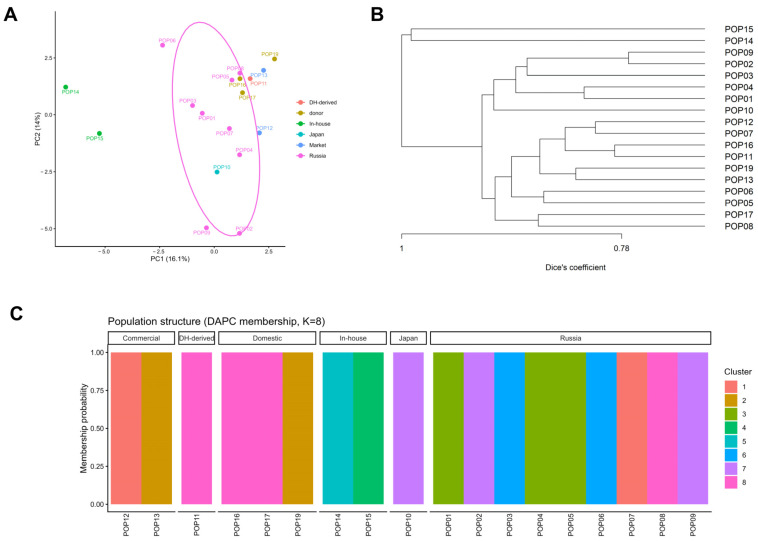
Genetic relationships of 18 popcorn inbred lines based on SSR markers. (**A**) PCA based on 25 popcorn-related SSR markers showing the genetic differentiation among popcorn lines. (**B**) UPGMA dendrogram constructed using Dice’s genetic distance, illustrating the clustering relationships among the materials. (**C**) Population structure analysis (K = 8) revealing multiple genetic components and varying levels of admixture among popcorn lines.

A total of 25 SSR markers were used for genetic analysis (Table 2). The number of alleles per locus ranged from 2 to 11, reflecting substantial allelic diversity among the tested lines. Expected heterozygosity (He) varied from 0.05 to 0.86, and PIC values ranged from 0.05 to 0.85. Notably, 10 markers exhibited PIC values greater than 0.50, indicating a high level of polymorphism. Among these, bnlg2077 (PIC = 0.85) and umc2163 (PIC = 0.74) showed particularly strong discriminatory capacity. Genetic relationships among the 18 popcorn inbred lines were further evaluated using PCA, UPGMA clustering, and population structure analysis based on these SSR markers (Figure 2). In the PCA (Figure 2A), the first two principal components explained 16.1% and 14.0% of the total genetic variation, respectively. The lines were broadly distributed across the PC1–PC2 plane, without forming a single compact cluster. Lines with similar breeding origins tended to occupy proximate regions, while others were more dispersed, suggesting heterogeneous genetic backgrounds within the germplasm collection. In the UPGMA dendrogram constructed using Dice’s coefficient (Figure 2B), the lines were divided into multiple distinct branches. The clustering pattern was largely consistent with the PCA results, indicating considerable genetic divergence among the inbred lines. Population structure analysis at K = 8 revealed that most lines were predominantly assigned to specific genetic clusters, whereas a few exhibited admixed genetic components (Figure 2C). These results together indicate the substantial genetic diversity within the studied popcorn germplasm.

### 2.5. Putative Marker–Trait Associations for Popping-Related Traits

**Figure 3:**
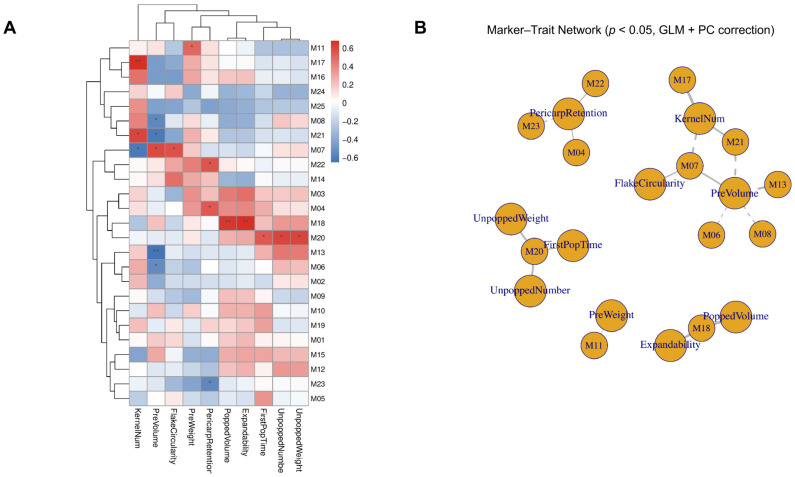
Association analysis between SSR markers and popping-related traits. (**A**) Correlation heatmap between SSR markers and popping-related traits, where * and ** indicate significance at *p* < 0.05 and *p* < 0.01, respectively. (**B**) Marker–trait association network at the locus level (*p* < 0.05, GLM + PC correction). Edge thickness is proportional to −log_10_(*p*).

Based on the confirmed genetic differentiation among the tested lines, we further explored associations between SSR markers and popping-related traits. The correlation heatmap (Figure 3A) revealed diverse association patterns between loci and phenotypic traits. The complete correlation coefficients and corresponding significance levels are provided in Table S4. Several markers exhibited positive or negative correlations across multiple traits, suggesting potential pleiotropic effects with functional genomic regions. Hierarchical clustering indicated that certain traits, such as expansion volume and expandability, formed closely related clusters. To further explore putative marker–trait associations, a marker–trait network was constructed based on general linear models (GLMs) analysis with principal component correction (*p* < 0.05) (Figure 3B). Multiple putative locus–trait associations were detected. Notably, marker M18 showed putative associations with both expandability and the expansion volume, indicating its potential role in expansion-related performance. In addition, several loci were associated with flake morphology-related traits, including flake circularity and pericarp retention, suggesting that post-expansion structural characteristics may be controlled by distinct genetic factors.

Overall, these results demonstrate that specific SSR loci are putatively associated with key popping-related traits, providing preliminary evidence for marker-assisted selection in popcorn breeding.

### 2.6. Integrated Model Linking SSR Loci and Popping Performance

**Figure 4:**
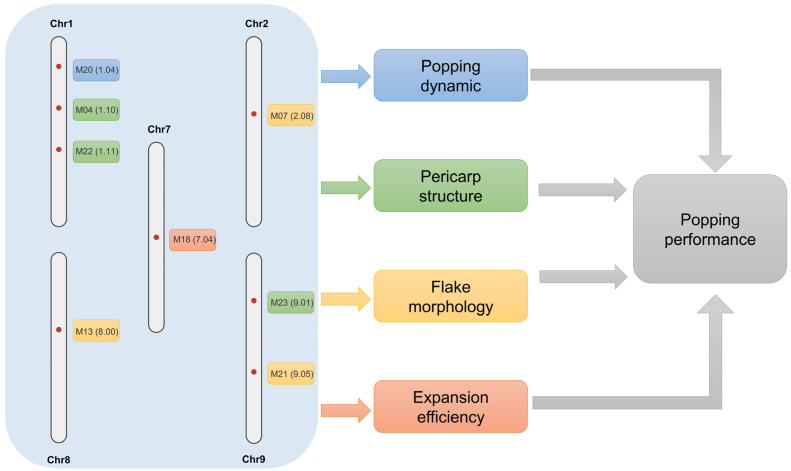
Proposed model linking SSR loci and popping performance.

To synthesize the association results, we propose an integrated model summarizing putative SSR loci to distinct functional components of popping performance (Figure 4). The putative loci detected in this study were distributed across multiple chromosomes rather than concentrated within a single genomic region, consistent with the complex genetic basis of popping-related traits. Based on the marker–trait associations, the loci could be categorized into four functional modules: popping dynamics, pericarp structure, flake morphology, and expansion efficiency. Specifically, M20 was predominantly associated with dynamic traits such as the first pop time, suggesting its potential involvement in regulating popping progression. Loci M04, M22 and M23 were linked to pericarp-related traits, particularly pericarp retention, implying a role in structural resistance during kernel expansion. Markers M07, M13, and M21 were associated with flake morphology-related traits, including circularity and post-expansion structural characteristics, indicating that flake-type variation may involve multiple genomic regions. In contrast, M18 exhibited putative associations with expansion efficiency-related indices such as expandability and the expansion volume, highlighting its potential contribution to overall popping performance.

Although these loci appear to influence different trait modules, their combined effects ultimately converge to determine final popping performance. This integrative framework emphasizes the modular yet interconnected nature of popcorn popping traits and provides a conceptual basis for future further validation and future marker development.

## 3. Discussion

Popcorn breeding in China has made progress in varietal development and quality evaluation, but the integration of quantitative phenotyping, digital flake shape analysis, and molecular marker information remains limited in breeding-oriented germplasm studies [3,19]. For quality-oriented popcorn improvement, combining popping-quality evaluation with SSR-based diversity and marker–trait analysis can provide practical information for identifying promising inbred lines, selecting genetically divergent parents, and designing crosses with improved popping performance [25]. In this study, we applied this integrated framework to a set of popcorn inbred lines to evaluate their phenotypic variation, genetic diversity and preliminary marker–trait associations.

Expansion volume and expandability are the core indicators of popcorn quality because they reflect the combined effects of other traits [19]. The phenotypic variation observed among the evaluated lines indicates that the studied germplasm contains useful diversity for improving popping performance (Table 1). Differences in first pop time and unpopped ratio may reflect genotypic variation in heat transfer efficiency, rupture threshold and kernel structural properties. Correlation analysis indicated that popping volume was positively associated with popping ratio and negatively associated with unpopped grain ratio, consistent with coordinated variation among popping-related traits [30]. The observed diversity is useful for breeding because it allows expansion efficiency and end-use quality to be considered simultaneously. Popcorn quality is not determined only by expansion, as processing methods substantially affect sensory attributes, starch behavior, and nutritional composition [31]; a practical evaluation system should integrate expansion, popping efficiency, flake characteristics, and end-use suitability together.

Flake morphology is another important determinant of popcorn quality and market differentiation [32]. Unilateral, bilateral, and multilateral flakes differ in packing behavior; therefore, flake shape variation influences the physical performance of the popped products [33]. Different flake polymorphisms are also linked to popping performance—unilateral flakes tend to show a negative correlation with expansion volume, whereas bilateral flakes tend to show a positive one [34]. Butterfly-type flakes typically have larger expansion areas and irregular protrusions, giving perceived volume and sensory lightness; mushroom-type flakes are more compact and spherical and are mechanically more stable during industrial tumbling, coating and packaging [2,5,35]. The predominance of butterfly flakes in our germplasm may reflect breeding preferences tied to direct-consumption markets [3]. Genotypes with a higher proportion of mushroom flakes suggest breeding potential for specialized industrial applications [7].

Traditional flake classification relies on categorical scoring, which can obscure intermediate phenotypes and reduce genetic resolution [6]. The circularity-based approach used here treated flake morphology as a continuous trait and showed that most kernels fell within intermediate circularity ranges rather than strictly binary classes (Figure 1). Flake morphology therefore appears to represents a continuum rather than a discrete classification. Objective digital phenotyping can reduce subjectivity and capture continuous variation, which should improve statistical power for downstream association analysis [36]. Digital morphology metrics link industrial quality assessment to quantitative genetic analysis [27].

SSR markers remain useful for evaluating genetic diversity and population structure in breeding materials owing to their polymorphism, reproducibility, and low cost [21,37,38,39]. The 25 SSR primer pairs used here, distributed across all 10 maize chromosomes, gave broad genome coverage and revealed clear genetic differentiation among the lines (Table 2). Moderate-to-high PIC values at several loci indicated sufficient allelic variation for downstream analysis. PCA, UPGMA clustering, and population structure analysis consistently showed heterogeneous backgrounds without a single dominant cluster (Figure 2), in line with previous reports that SSR markers resolve genetic structure and divergence among popcorn accessions [24]. The population was structured but not severely stratified, which should reduce confounding and help the interpretation of the association analysis [40]. Populations with high heterozygosity and genetic diversity can serve as reservoirs for inbred line development and for broadening the genetic base of popcorn breeding programs [41]. Diversity information of this kind is also useful for selecting genetically divergent parents and for designing crosses that maximize the chance of recovering useful recombination.

Population structure was incorporated into the GLM (Figure 3), and the observed genetic heterogeneity likely increased power to detect multi-locus effects. The marker–trait association results indicate that popping-related traits fall into several functional groups that interact but are not fully dependent on each other. Loci associated with expansion efficiency were largely separate from those linked to flake morphology or pericarp traits, consistent with the view that different biological processes contribute to different components of popping performance. Popcorn quality therefore appears to depend on coordinated effects across multiple trait groups rather than on a single major-effect locus [42], matching previous genetic studies that describe popping-related traits as quantitatively inherited, subject to genotype-by-environment interaction, and influenced by multiple genomic regions [26].

Expansion efficiency depends on a sequence of tightly regulated biological steps, including starch gelatinization [43], steam pressure accumulation [8], protein composition [11], and pericarp rupture [10]. Variation in starch composition, the amylose-to-amylopectin ratio, and kernel vitreousness can alter internal pressure dynamics, while pericarp thickness and structural integrity control rupture timing and expansion symmetry [1,44]. The detection of putative loci associated with flake circularity and pericarp retention is consistent with the hypothesis that post-expansion structural traits are at least partly separable from volumetric expansion traits [1]. That separability matters practically because breeders may be able to improve expandability while maintaining a desired flake type, or alter flake morphology without substantial losses in expansion volume. Trait decoupling of this kind is particularly relevant for diversified industrial markets that require both high expansion efficiency and structural robustness [32].

Several of the associated loci identified here lie in chromosomal regions previously implicated in kernel structure and starch-related traits, which supports the biological plausibility of the signals (Figure 4). Earlier QTL and association studies have shown that expansion-related traits are distributed across multiple maize chromosomes, consistent with a polygenic architecture [18,45]. Chromosome 1 was again identified here as an important genomic region for popping volume [46,47]. The loci on chromosomes 2 and 9 detected in our study also show chromosome-level agreement with previous reports [48], suggesting that these chromosomes harbor regions of recurrent importance for popping-related traits. Our interpretation of pericarp-related loci is consistent with earlier evidence that pericarp thickness is controlled by multiple loci distributed across the maize genome [49]. Gong et al. [50] proposed that pericarp thickness variation is largely determined by pericarp cell thickness and mesocarp cell-layer dynamics, giving a structural basis for how allelic differences in the kernel’s outer layers might alter rupture behavior during heating.

Earlier work has suggested that the clustering of commercial popcorn materials into a limited genetic group may indicate a narrow genetic base, emphasizing the need to introduce and exploit more diverse germplasm [24]. SSR-based diversity analysis, beyond describing population structure, helps identify genetically divergent germplasm and aids parent selection for hybrid breeding and genetic base broadening. The diversity patterns detected here thus matter for interpreting the current association results and for guiding future germplasm use and population construction.

Although this study provides an integrated evaluation of popping quality, flake morphology, SSR-based genetic diversity, and preliminary marker–trait associations in popcorn inbred lines, several limitations should be acknowledged. First, the population size was relatively limited. The inbred lines used in this study were selected from the popcorn germplasm currently available in our breeding program, and the number of lines was further constrained by seed availability and field establishment. Therefore, the detected phenotypic variation and marker–trait associations should be interpreted as preliminary signals rather than definitive loci. Nevertheless, these SSR-level signals are useful for identifying promising genomic regions and trait modules that can be prioritized in subsequent breeding studies [51]. Future studies should include larger and more diverse germplasm panels, as well as segregate populations derived from contrasting parental combinations, to validate the stability and breeding value of these associations.

Second, although the 25 SSR markers used in this study were selected from published maize- and popcorn-related studies and distributed across the 10 maize chromosomes, their density remains moderate compared with high-throughput SNP-based genotyping platforms. SSR markers are still useful for low-cost diversity assessment and preliminary screening, but they have limited resolution for fine mapping and marker-assisted selection. In addition, several SSR loci showed relatively high heterozygosity, suggesting that further purification and genetic stabilization of some inbred lines may be necessary before their direct use in breeding or validation populations. High-density SNP genotyping, BSA, and KASP marker development could then be used to refine these preliminary signals and convert them into more robust tools for marker-assisted selection [52].

Finally, several phenotyping and validation procedures should be further improved in future work. Because seeds from inbred lines were limited, a 10 g popping sample was used instead of the larger sample size recommended in CIMMYT standard protocols. Although this allowed for consistent comparison among scarce breeding materials, future studies should increase sample size when seed quantity allows and compare results with standardized popping procedures. Moreover, although the ImageJ-based circularity analysis provided an objective and continuous description of flake morphology, the classification thresholds should be further validated using manual scoring, industrial quality evaluation, or consumer-oriented criteria. In parallel, transcriptomic, cytological, or biochemical analyses of kernel structure, pericarp characteristics, and starch–protein composition would help clarify the biological basis of the trait modules proposed here. These methodological improvements and follow-up analyses would strengthen the connection between digital phenotyping, biological interpretation, and practical popcorn breeding.

## 4. Materials and Methods

### 4.1. Plant Materials and Field Experiments

**Figure 5:**
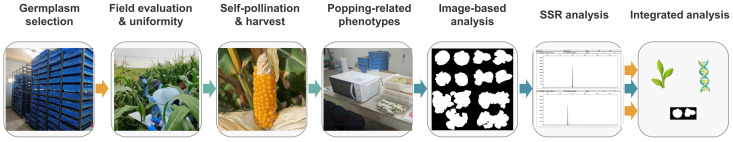
Experimental workflow for phenotypic and molecular evaluation of popcorn inbred lines.

A total of 20 popcorn inbred lines were initially selected as parental candidates for germplasm evaluation and quality improvement in this study. These inbred lines were developed and maintained by the Maize Research Institute, Liaoning Academy of Agricultural Sciences, Shenyang, Liaoning, China. The materials represented diverse genetic backgrounds within our in-house popcorn breeding program. Our institute previously released a popcorn variety, Liaobao No. 1, which has provided a breeding foundation for improving popcorn in China. Based on this foundation, additional in-house popcorn germplasm resources were systematically organized and evaluated to identify superior parental lines and support the development of improved popcorn cultivars with enhanced popping performance and quality traits (Figure 5).

Field experiments were conducted in Shenyang, Liaoning Province, China, during the spring growing season of 2025. All materials were planted in two adjacent rows with 12 plants per row, using a row spacing of 0.60 m and a plant spacing of 0.25 m, for field evaluation. The experimental field was arranged in a completely randomized design under standard field management practices.

After field establishment, two lines failed to germinate and were excluded. Among the remaining 18 lines, 16 lines with sufficient seed quantity were used for popping-related phenotypic evaluation, whereas all 18 lines were retained for SSR-based genetic analysis.

### 4.2. Evaluation of Popping-Related Traits

Popping-related traits were evaluated following the standardized protocol recommended by the International Maize and Wheat Improvement Center (CIMMYT), with minor modifications [53]. All kernels were harvested at physiological maturity and equilibrated to a uniform moisture content of approximately 13% prior to popping tests to minimize moisture-related variation. In the original CIMMYT protocol, 30 g kernels are used for each popping test and popped in a microwave oven under a standardized program. In the present study, because seed quantities of some inbred lines were limited, the sample size was reduced to 10 g per replicate. Kernels were popped using a microwave oven (Galanz, SE model, Foshan, China) at 700 W for 2 min. The expansion volume was recorded as the final volume of the popped sample, and expandability was calculated as the ratio of popped volume to the pre-popping kernel volume and is expressed as fold expansion.

### 4.3. Image-Based Flake Morphology Analysis

Flake morphology was quantitatively evaluated using an image-based phenotyping approach to characterize shape variation among popped kernels. Individual flakes were placed on a black background, and images were captured under standardized lighting and distance conditions to minimize environmental variation.

Image processing and morphological analysis were performed using the ImageJ software (version 1.53t, National Institutes of Health, Bethesda, MD, USA). Individual flakes were manually segmented from the background and converted into binary images prior to feature extraction. Flake shape was quantitatively described using the circularity index, with higher circularity values indicating shapes approaching a typical mushroom-type popcorn flake.

Flake shape classes were assigned using an operational classification scheme based on the flake polymorphism categories described by Sweley et al. [6], combined with visual inspection and repeated comparison between manually recognized flake types and their corresponding circularity values. Based on this calibration procedure, the following circularity thresholds were used in this study: mushroom-shaped flakes, 0.67–1.00; unilaterally expanded butterfly flakes, 0.48–0.67; multilaterally expanded butterfly flakes, 0.40–0.48; and bilaterally expanded butterfly flakes, 0.32–0.40. This classification was used as an image-based operational framework for quantitative analysis rather than as an industrial grading standard.

### 4.4. DNA Extraction and SSR Genotyping

Genomic DNA was extracted from young leaf tissue using a commercial plant DNA extraction kit (D200, GeneBetter Biotech, Beijing, China) following the manufacturer’s instructions. DNA quality and concentration were assessed using a NanoDrop spectrophotometer (Thermo Fisher Scientific, Waltham, MA, USA), and all DNA samples were diluted to a uniform working concentration of 5 ng/µL prior to PCR amplification.

SSR markers were selected based on previous studies and published reports related to maize genetic diversity and popping-related traits [23,24,30,38,39,41]. A total of 25 SSR primer pairs distributed across all 10 maize chromosomes were used in this study. PCR amplification was performed following standard procedures, with annealing temperatures optimized according to each primer’s Tm value. To enable high-throughput genotyping, four fluorescent dyes (FAM, HEX, TAMRA, and ROX) were used in combination with an 8× multiplex pooling strategy. Amplified products were detected by capillary electrophoresis.

### 4.5. Genetic Structure Analysis

PCA was performed to further evaluate genetic differentiation and population structures among the inbred lines. In addition, the population structure was inferred to identify subpopulation composition and genetic ancestry.

Genetic relationships among the 18 popcorn inbred lines were assessed based on SSR genotyping data. SSR allele data were converted into genotype format and then transformed into a genind object using the adegenet package in R software (version 4.5.2; R Foundation for Statistical Computing, Vienna, Austria). Nei’s genetic distance was calculated using the poppr package, and an unweighted pair group method with an arithmetic mean (UPGMA) dendrogram was constructed using the average-linkage clustering method. PCA was performed using allele count data extracted from the genind object to further evaluate genetic differentiation among the inbred lines.

To provide an exploratory visualization of genetic cluster membership, the SSR-based clustering pattern was further summarized using a DAPC-style membership plot. Because of the limited number of inbred lines, the number of displayed clusters was set to K = 8 to match the major groups observed in the SSR-based clustering and source-related differentiation.

### 4.6. Marker–Trait Association Analysis

Marker–trait association analysis was performed to explore relationships between SSR loci and popping-related traits. The mean phenotypic values of each inbred line were used for the analysis. For each SSR marker, allelic variation was coded as the binary presence/absence of data and tested against trait values using GLMs in R. Data handling was performed using the tidyverse package, and GLMs were fitted using the lm function in base R. To reduce the influence of population structure, principal component (PC) covariates derived from the SSR dataset were included in the model. The following model was used: trait value = marker effect + PC1 + PC2 + residual error. A total of 385 marker–trait tests were performed in this study. Marker–trait associations with *p* < 0.05 were considered putatively significant. To account for multiple testing, *p*-values were further adjusted within each trait using the Benjamini–Hochberg false discovery rate (FDR) procedure implemented with the p.adjust function in R. The association results were summarized and visualized using heatmaps and network plots to illustrate the relationships between markers and traits.

### 4.7. Statistical Analysis

Allele sizes were determined based on capillary electrophoresis profiles and analyzed using GeneMarker software (version 1.65; SoftGenetics LLC, State College, PA, USA). All phenotypic data were analyzed using R software. Analysis of variance (ANOVA) was performed to evaluate differences among popcorn inbred lines for popping-related traits. Pearson correlation analysis was conducted to assess relationships among traits. Statistical significance was determined at *p* < 0.05. Data visualization was performed using R packages specified above and Microsoft Excel 2019 (Microsoft Corporation, Redmond, WA, USA).

## 5. Conclusions

This study evaluated popping-related phenotypic variation, genetic diversity, and marker–trait relationships in popcorn inbred lines. The tested materials showed considerable variation in expansion-related traits, popping dynamics, pericarp retention and flake morphology, indicating their potential value for quality-oriented germplasm evaluation and parental selection. The image-based phenotyping approach demonstrated that flake morphology can be quantified as a continuous trait, providing a more objective and informative complement to conventional categorical scoring.

SSR analysis revealed clear genetic differentiation and moderate-to-high polymorphism across the evaluated lines, indicating that the selected marker set is useful for diversity analysis in popcorn breeding materials. Exploratory marker–trait association analysis identified several putatively significant loci linked to distinct trait components, including expansion efficiency, flake morphology, pericarp-related characteristics, and popping dynamics. These results support the view that popping performance may controlled by multiple partially independent but interconnected trait modules rather than by a single major-effect factor.

Overall, this study demonstrates the usefulness of SSR markers combined with quantitative phenotyping for characterizing popcorn germplasm and exploring the genetic basis of popping-related traits. Although further validation using larger populations and high-density markers is required, the identified genetic variation, population structure, and putative marker–trait associations provide a useful foundation for marker-assisted popcorn improvement.

## Figures and Tables

**Table 1:** Popping phenotypic performance of 16 popcorn inbred lines.

Line	EV (mL)	Expandability (Fold)	First Pop Time (s)	UR (%)	PR (Mean)	Mushroom (%)
POP01	276.67 ± 11.55	25.86 ± 1.24	31.00 ± 7.21	0 ± 0	2.2	0
POP02	340.00 ± 26.46	32.27 ± 1.61	44.00 ± 8.00	0 ± 0	3.4	0
POP03	263.33 ± 5.77	24.75 ± 0.54	42.33 ± 2.52	3.40 ± 3.03	2.2	0
POP04	310.00 ± 10.00	29.42 ± 0.74	51.67 ± 4.16	1.46 ± 1.45	2.0	0
POP05	283.33 ± 11.55	27.52 ± 1.05	41.33 ± 8.33	2.76 ± 3.48	2.0	40
POP06	316.67 ± 25.17	29.45 ± 2.51	48.33 ± 5.69	1.32 ± 2.28	3.2	0
POP07	343.33 ± 28.87	32.46 ± 1.60	45.67 ± 2.08	1.11 ± 1.92	4.0	0
POP08	193.33 ± 15.28	18.60 ± 1.65	39.33 ± 4.16	0 ± 0	2.7	0
POP09	306.67 ± 45.09	29.10 ± 3.13	32.33 ± 3.06	0.37 ± 0.64	2.0	0
POP10	206.67 ± 30.55	20.36 ± 2.97	39.67 ± 2.08	0 ± 0	2.0	0
POP11	326.67 ± 25.17	31.23 ± 2.94	39.33 ± 5.69	0.65 ± 1.13	2.0	0
POP12	313.33 ± 20.82	28.84 ± 1.98	41.00 ± 6.24	0 ± 0	2.0	0
POP13	173.33 ± 15.28	16.79 ± 1.46	43.67 ± 5.03	0.71 ± 1.23	1.1	10
POP14	193.33 ± 5.77	17.81 ± 0.39	37.00 ± 5.29	0 ± 0	3.9	10
POP15	173.33 ± 5.77	16.84 ± 0.83	38.67 ± 2.08	0 ± 0	1.0	10
POP19	240.00 ± 0	22.67 ± 0.16	40.33 ± 2.52	0 ± 0	3.4	0

Data are presented as mean ± standard deviation (SD). EV, expansion volume; UR, unpopped ratio; PR, pericarp retention.

**Table 2:** Summary statistics of the 25 SSR markers used in this study.

No.	Marker Name	Bin	Repeat Motif	Product Size (Min–Max)	Na	Ho	He	PIC
M01	bnlg2077	2.07	(AG)_33_	105–135	11	0.83	0.86	0.85
M02	umc2163	10.04	(AG)_28_	75–164	7	0.33	0.77	0.74
M03	umc1019	5.06	(CT)_17_	78–100	5	0.39	0.73	0.68
M04	umc2189	1.10	(CAG)_4_	104–141	6	0.78	0.72	0.67
M05	umc1155	5.05	(AG)_20_	129–162	4	0	0.66	0.60
M06	phi029	3.04	AG/AGCG	94–158	5	1.00	0.65	0.58
M07	phi127	2.08	AGAC	101–124	5	0	0.63	0.57
M08	umc1327	8.01	(GCC)_4_	75–81	6	0.11	0.59	0.54
M09	umc1841	7.03	(CGG)_6_	100–108	3	0.17	0.61	0.54
M10	umc1936	7.03	(TG)_8_	82–162	5	0.06	0.59	0.51
M11	umc2012	1.01	-	75–98	4	0	0.54	0.48
M12	umc1148	3.07	(GA)_12_	147–174	4	0.06	0.54	0.47
M13	phi420701	8.00	CCG	163–297	4	0.17	0.51	0.46
M14	umc1299	4.06	(AAG)_5_	139–152	3	0.06	0.54	0.45
M15	umc1329	4.06	(GCC)_7_	82–164	3	1.00	0.53	0.41
M16	umc1296	6.06	(GGT)_7_	77–138	3	0.33	0.43	0.39
M17	phi299852	6.07	AGC	108–120	2	0.17	0.49	0.37
M18	phi328175	7.04	AGG	98–125	4	0.06	0.33	0.31
M19	umc1061	10.06	(TCG)_6_	100–107	3	0.06	0.36	0.31
M20	bnlg2086	1.04	(AG)_18_	231–233	2	0	0.28	0.24
M21	umc1357	9.05	(CTG)_8_	130–132	2	0	0.28	0.24
M22	umc1118	1.11	(GAGCA)_4_	114–146	3	0	0.20	0.19
M23	phi033	9.01	AAG	243–254	3	0	0.20	0.19
M24	umc1186	6.01	(GCT)_5_	209–216	2	0.06	0.15	0.14
M25	umc1071	1.01	(TACGA)_5_	87–128	2	0.06	0.05	0.05

Na = number of alleles; Ho = observed heterozygosity; He = expected heterozygosity; PIC = polymorphism information content.

## Data Availability

The original contributions presented in this study are included in the article/Supplementary Material. Further inquiries can be directed to the corresponding authors.

## Supplementary Materials

The following supporting information can be downloaded at https://www.mdpi.com/article/10.3390/genes17060690/s1, Figure S1: Uniformity evaluation of popcorn inbred lines; Table S1: List of popcorn inbred lines used in this study; Table S2: Raw phenotypic data of popping-related traits; Table S3: Additional phenotypic measurements; Table S4: Correlation coefficients between SSR markers and traits.
