# Supplementary material for: SSR-Based Genetic Diversity, Population Structure, and Marker–Trait Associations for Popping-Related Traits in Popcorn Germplasm
Source: Genes (Basel). 2026 Jun 12;17(6):690. doi: 10.3390/genes17060690 (PMC13300098; doi:10.3390/genes17060690)
Supplement: Supplementary file 1 [file genes-17-00690-s001.zip › List.pdf]

List of Supplementary Materials

Figure S1. Uniformity evaluation of popcorn inbred lines.
Table S1. List of popcorn inbred lines used in this study.
Table S2. Raw phenotypic data of popping-related traits.
Table S3. Additional phenotypic measurements.
Table S4. Correlation coefficients between SSR markers and traits.
