# Supplementary material for: SSR-Based Genetic Diversity, Population Structure, and Marker–Trait Associations for Popping-Related Traits in Popcorn Germplasm
Source: Genes (Basel). 2026 Jun 12;17(6):690. doi: 10.3390/genes17060690 (PMC13300098; doi:10.3390/genes17060690)
Supplement: Supplementary file 1 [file genes-17-00690-s001.zip › Table_S1.pdf]

Table S1. List of popcorn inbred lines used in this study.

ID	Overall score	Source	Note
POP14	4.75	In-house	
POP16	4.25	Domestic	Excluded
POP05	4.00	Russia	
POP09	4.00	Russia	
POP10	4.00	Japan	
POP11	4.00	DH-derived	
POP08	3.75	Russia	
POP12	3.75	Commercial	
POP17	3.75	Domestic	Excluded
POP19	3.75	Domestic	
POP06	3.50	Russia	
POP07	3.50	Russia	
POP04	3.25	Russia	
POP13	3.25	Commercial	
POP02	3.00	Russia	
POP01	2.50	Russia	
POP15	2.50	In-house	
POP03	2.25	Russia	
POP18	0	Domestic	Failed to germinate
POP20	0	In-house	Failed to germinate
