# Supplementary material for: SSR-Based Genetic Diversity, Population Structure, and Marker–Trait Associations for Popping-Related Traits in Popcorn Germplasm
Source: Genes (Basel). 2026 Jun 12;17(6):690. doi: 10.3390/genes17060690 (PMC13300098; doi:10.3390/genes17060690)
Supplement: Supplementary file 1 [file genes-17-00690-s001.zip › Table_S2.pdf]

Table S2. Raw phenotypic data of popping-related traits.

Line	Replicate	Kernel Number	PreWeight (g)	PreVolume (mL)	FirstPop Time(s)	Grain Color	Flake Color	Popped Volume(mL)	Unpopped Number	Unpopped Weight(g)	Expandability
POP01	1	67	10.60	16.00	25	YELLOW	CREAM	270.00	0	0	25.12
POP01	2	65	10.58	16.00	39	YELLOW	CREAM	270.00	0	0	25.17
POP01	3	64	10.48	15.00	29	YELLOW	CREAM	290.00	0	0	27.29
POP02	1	81	10.21	14.00	44	YELLOW	CREAM	330.00	0	0	31.50
POP02	2	78	10.00	13.00	52	YELLOW	CREAM	320.00	0	0	31.19
POP02	3	80	10.57	15.00	36	YELLOW	CREAM	370.00	0	0	34.12
POP03	1	68	10.45	14.00	45	YELLOW	CREAM	270.00	3	0.48	25.33
POP03	2	69	10.51	14.00	40	YELLOW	CREAM	260.00	4	0.58	24.25
POP03	3	69	10.34	14.00	42	YELLOW	CREAM	260.00	0	0	24.65
POP04	1	68	10.44	15.00	55	YELLOW	CREAM	300.00	1	0.14	28.70
POP04	2	70	10.54	15.00	47	YELLOW	CREAM	310.00	0	0	29.38
POP04	3	69	10.59	15.00	53	YELLOW	CREAM	320.00	2	0.23	30.18
POP05	1	62	10.09	14.00	32	YELLOW	CREAM	290.00	1	0.13	28.58
POP05	2	60	10.14	15.00	48	YELLOW	CREAM	270.00	4	0.42	26.47
POP05	3	62	10.48	14.00	44	YELLOW	CREAM	290.00	0	0	27.51
POP06	1	78	10.41	14.00	50	YELLOW	CREAM	340.00	0	0	32.14
POP06	2	80	10.84	14.00	42	YELLOW	CREAM	320.00	0	0	29.05
POP06	3	76	10.50	14.00	53	YELLOW	CREAM	290.00	3	0.28	27.17
POP07	1	60	10.11	15.00	48	YELLOW	CREAM	310.00	2	0.26	30.63
POP07	2	62	10.70	15.50	44	YELLOW	CREAM	360.00	0	0	33.61
POP07	3	62	10.85	15.00	45	YELLOW	CREAM	360.00	0	0	33.14
POP08	1	87	10.18	15.00	36	YELLOW	CREAM	210.00	0	0	20.51
POP08	2	71	10.16	14.00	44	YELLOW	CREAM	180.00	0	0	17.61
POP08	3	75	10.68	14.00	38	YELLOW	CREAM	190.00	0	0	17.69
POP09	1	83	10.03	13.50	29	YELLOW	CREAM	260.00	0	0	25.77
POP09	2	90	10.88	15.00	35	YELLOW	CREAM	350.00	1	0.09	31.98
POP09	3	87	10.43	14.00	33	YELLOW	CREAM	310.00	0	0	29.55
POP10	1	84	10.10	14.50	39	YELLOW	CREAM	240.00	0	0	23.63
POP10	2	71	10.04	14.50	42	YELLOW	CREAM	180.00	0	0	17.83
POP10	3	68	10.13	15.00	38	YELLOW	CREAM	200.00	0	0	19.63
POP11	1	51	10.11	14.50	44	YELLOW	CREAM	350.00	1	0.23	33.98
POP11	2	47	10.26	15.00	41	YELLOW	CREAM	330.00	0	0	31.57

Table S2. Raw phenotypic data of popping-related traits.

Line	Replicate	Kernel Number	PreWeight (g)	PreVolume (mL)	FirstPop Time(s)	Grain Color	Flake Color	Popped Volume(mL)	Unpopped Number	Unpopped Weight(g)	Expandability
POP11	3	48	10.47	15.50	33	YELLOW	CREAM	300.00	0	0	28.13
POP12	1	64	10.65	15.00	46	YELLOW	CREAM	330.00	0	0	30.31
POP12	2	58	10.67	15.00	34	YELLOW	CREAM	290.00	0	0	26.59
POP12	3	56	10.57	15.00	43	YELLOW	CREAM	320.00	0	0	29.61
POP13	1	53	10.02	14.50	43	YELLOW	CREAM	170.00	0	0	16.62
POP13	2	49	10.15	15.00	49	YELLOW	CREAM	190.00	0	0	18.33
POP13	3	47	10.15	15.00	39	YELLOW	CREAM	160.00	1	0.19	15.44
POP14	1	292	10.52	14.00	43	WHITE	WHITE	190.00	0	0	17.62
POP14	2	209	10.69	14.00	33	WHITE	WHITE	200.00	0	0	18.26
POP14	3	181	10.57	14.00	35	WHITE	WHITE	190.00	0	0	17.54
POP15	1	189	10.06	13.50	41	RED	WHITE	180.00	0	0	17.79
POP15	2	139	10.26	13.00	37	RED	WHITE	170.00	0	0	16.47
POP15	3	150	10.40	14.00	38	RED	WHITE	170.00	0	0	16.25
POP19	1	77	10.37	15.00	40	YELLOW	CREAM	240.00	0	0	22.61
POP19	2	70	10.40	15.00	38	YELLOW	CREAM	240.00	0	0	22.55
POP19	3	67	10.26	15.00	43	YELLOW	CREAM	240.00	0	0	22.85
