# Supplementary material for: SSR-Based Genetic Diversity, Population Structure, and Marker–Trait Associations for Popping-Related Traits in Popcorn Germplasm
Source: Genes (Basel). 2026 Jun 12;17(6):690. doi: 10.3390/genes17060690 (PMC13300098; doi:10.3390/genes17060690)
Supplement: Supplementary file 1 [file genes-17-00690-s001.zip › Table_S3.pdf]

Table S3. Additional phenotypic measurements.

Line	Flake_ID	Cir.	FS_class	PR_class
POP01	1	0.528	Unilaterally expanded	2
POP01	2	0.610	Unilaterally expanded	2
POP01	3	0.456	Bilaterally expanded	2
POP01	4	0.433	Bilaterally expanded	3
POP01	5	0.411	Bilaterally expanded	2
POP01	6	0.454	Bilaterally expanded	2
POP01	7	0.616	Unilaterally expanded	3
POP01	8	0.642	Unilaterally expanded	2
POP01	9	0.418	Bilaterally expanded	2
POP01	10	0.395	Multilaterally expanded	2
POP02	1	0.312	Multilaterally expanded	4
POP02	2	0.456	Bilaterally expanded	3
POP02	3	0.454	Bilaterally expanded	3
POP02	4	0.262	Multilaterally expanded	4
POP02	5	0.455	Bilaterally expanded	3
POP02	6	0.550	Unilaterally expanded	4
POP02	7	0.464	Bilaterally expanded	3
POP02	8	0.439	Bilaterally expanded	3
POP02	9	0.348	Multilaterally expanded	4
POP02	10	0.348	Multilaterally expanded	3
POP03	1	0.650	Unilaterally expanded	2
POP03	2	0.462	Bilaterally expanded	2
POP03	3	0.334	Multilaterally expanded	2
POP03	4	0.385	Multilaterally expanded	3
POP03	5	0.516	Unilaterally expanded	2
POP03	6	0.543	Unilaterally expanded	2
POP03	7	0.511	Unilaterally expanded	2
POP03	8	0.456	Bilaterally expanded	3
POP03	9	0.481	Unilaterally expanded	2
POP03	10	0.589	Unilaterally expanded	2
POP04	1	0.487	Unilaterally expanded	2
POP04	2	0.404	Bilaterally expanded	2
POP04	3	0.408	Bilaterally expanded	2
POP04	4	0.579	Unilaterally expanded	2
POP04	5	0.530	Unilaterally expanded	2
POP04	6	0.106	Multilaterally expanded	2
POP04	7	0.312	Multilaterally expanded	2
POP04	8	0.574	Unilaterally expanded	2
POP04	9	0.454	Bilaterally expanded	2
POP04	10	0.420	Bilaterally expanded	2
POP05	1	0.415	Bilaterally expanded	2
POP05	2	0.348	Multilaterally expanded	2
POP05	3	0.478	Bilaterally expanded	2
POP05	4	0.392	Multilaterally expanded	2
POP05	5	0.556	Unilaterally expanded	2
POP05	6	0.282	Multilaterally expanded	2
POP05	7	0.495	Unilaterally expanded	2
POP05	8	0.449	Bilaterally expanded	2
POP05	9	0.617	Unilaterally expanded	2
POP05	10	0.488	Unilaterally expanded	2
POP06	1	0.354	Multilaterally expanded	3
POP06	2	0.692	Mushroom	3
POP06	3	0.315	Multilaterally expanded	3
POP06	4	0.300	Multilaterally expanded	3
POP06	5	0.563	Unilaterally expanded	3
POP06	6	0.345	Multilaterally expanded	3
POP06	7	0.473	Bilaterally expanded	3
POP06	8	0.321	Multilaterally expanded	3

Table S3. Additional phenotypic measurements.

Line	Flake_ID	Cir.	FS_class	PR_class
POP06	9	0.335	Multilaterally expanded	4
POP06	10	0.386	Multilaterally expanded	4
POP07	1	0.463	Bilaterally expanded	4
POP07	2	0.393	Multilaterally expanded	4
POP07	3	0.384	Multilaterally expanded	4
POP07	4	0.251	Multilaterally expanded	4
POP07	5	0.490	Unilaterally expanded	4
POP07	6	0.433	Bilaterally expanded	4
POP07	7	0.318	Multilaterally expanded	4
POP07	8	0.545	Unilaterally expanded	4
POP07	9	0.716	Mushroom	4
POP07	10	0.425	Bilaterally expanded	4
POP08	1	0.770	Mushroom	3
POP08	2	0.564	Unilaterally expanded	3
POP08	3	0.540	Unilaterally expanded	2
POP08	4	0.540	Unilaterally expanded	3
POP08	5	0.630	Unilaterally expanded	3
POP08	6	0.295	Multilaterally expanded	2
POP08	7	0.313	Multilaterally expanded	3
POP08	8	0.559	Unilaterally expanded	3
POP08	9	0.405	Bilaterally expanded	3
POP08	10	0.606	Unilaterally expanded	2
POP09	1	0.457	Bilaterally expanded	2
POP09	2	0.524	Unilaterally expanded	2
POP09	3	0.138	Multilaterally expanded	2
POP09	4	0.191	Multilaterally expanded	2
POP09	5	0.226	Multilaterally expanded	2
POP09	6	0.356	Multilaterally expanded	2
POP09	7	0.326	Multilaterally expanded	2
POP09	8	0.389	Multilaterally expanded	2
POP09	9	0.499	Unilaterally expanded	2
POP09	10	0.501	Unilaterally expanded	2
POP10	1	0.493	Unilaterally expanded	2
POP10	2	0.539	Unilaterally expanded	2
POP10	3	0.388	Multilaterally expanded	2
POP10	4	0.426	Bilaterally expanded	2
POP10	5	0.516	Unilaterally expanded	2
POP10	6	0.426	Bilaterally expanded	2
POP10	7	0.337	Multilaterally expanded	2
POP10	8	0.175	Multilaterally expanded	2
POP10	9	0.463	Bilaterally expanded	2
POP10	10	0.500	Unilaterally expanded	2
POP11	1	0.438	Bilaterally expanded	2
POP11	2	0.426	Bilaterally expanded	2
POP11	3	0.093	Multilaterally expanded	2
POP11	4	0.401	Bilaterally expanded	2
POP11	5	0.521	Unilaterally expanded	2
POP11	6	0.546	Unilaterally expanded	2
POP11	7	0.527	Unilaterally expanded	2
POP11	8	0.347	Multilaterally expanded	2
POP11	9	0.443	Bilaterally expanded	2
POP11	10	0.427	Bilaterally expanded	2
POP12	1	0.358	Multilaterally expanded	2
POP12	2	0.320	Multilaterally expanded	2
POP12	3	0.290	Multilaterally expanded	2
POP12	4	0.224	Multilaterally expanded	2
POP12	5	0.382	Multilaterally expanded	2
POP12	6	0.446	Bilaterally expanded	2

Table S3. Additional phenotypic measurements.

Line	Flake_ID	Cir.	FS_class	PR_class
POP12	7	0.191	Multilaterally expanded	2
POP12	8	0.459	Bilaterally expanded	2
POP12	9	0.573	Unilaterally expanded	2
POP12	10	0.503	Unilaterally expanded	2
POP13	1	0.777	Mushroom	1
POP13	2	0.473	Bilaterally expanded	1
POP13	3	0.803	Mushroom	1
POP13	4	0.616	Unilaterally expanded	1
POP13	5	0.416	Bilaterally expanded	1
POP13	6	0.760	Mushroom	1
POP13	7	0.517	Unilaterally expanded	1
POP13	8	0.536	Unilaterally expanded	1
POP13	9	0.450	Bilaterally expanded	2
POP13	10	0.763	Mushroom	1
POP14	1	0.410	Bilaterally expanded	3
POP14	2	0.527	Unilaterally expanded	4
POP14	3	0.382	Multilaterally expanded	4
POP14	4	0.451	Bilaterally expanded	4
POP14	5	0.472	Bilaterally expanded	4
POP14	6	0.600	Unilaterally expanded	4
POP14	7	0.505	Unilaterally expanded	4
POP14	8	0.352	Multilaterally expanded	4
POP14	9	0.275	Multilaterally expanded	4
POP14	10	0.379	Multilaterally expanded	4
POP15	1	0.569	Unilaterally expanded	1
POP15	2	0.457	Bilaterally expanded	1
POP15	3	0.500	Unilaterally expanded	1
POP15	4	0.127	Multilaterally expanded	1
POP15	5	0.306	Multilaterally expanded	1
POP15	6	0.398	Multilaterally expanded	1
POP15	7	0.207	Multilaterally expanded	1
POP15	8	0.180	Multilaterally expanded	1
POP15	9	0.451	Bilaterally expanded	1
POP15	10	0.150	Multilaterally expanded	1
POP19	1	0.570	Unilaterally expanded	4
POP19	2	0.636	Unilaterally expanded	3
POP19	3	0.224	Multilaterally expanded	3
POP19	4	0.537	Unilaterally expanded	4
POP19	5	0.492	Unilaterally expanded	3
POP19	6	0.557	Unilaterally expanded	4
POP19	7	0.496	Unilaterally expanded	4
POP19	8	0.490	Unilaterally expanded	3
POP19	9	0.417	Bilaterally expanded	3
POP19	10	0.455	Bilaterally expanded	3
