# Supplementary material for: SSR-Based Genetic Diversity, Population Structure, and Marker–Trait Associations for Popping-Related Traits in Popcorn Germplasm
Source: Genes (Basel). 2026 Jun 12;17(6):690. doi: 10.3390/genes17060690 (PMC13300098; doi:10.3390/genes17060690)
Supplement: Supplementary file 1 [file genes-17-00690-s001.zip › Table_S4.pdf]

Table S4. Correlation coefficients between SSR markers and traits.

Marker	Trait	Beta	*p*-value
M1_1	FirstPopTime	1.157694315	0.456463592
M1_2	FirstPopTime	0.248525438	0.798030028
M2_1	FirstPopTime	-0.078577966	0.746671227
M2_2	FirstPopTime	-0.008254356	0.976391222
M3_1	FirstPopTime	0.738435538	0.213916253
M3_2	FirstPopTime	0.711372905	0.281225294
M4_2	FirstPopTime	-0.274417978	0.358065566
M5_1	FirstPopTime	0.003490961	0.995752532
M5_2	FirstPopTime	0.003490961	0.995752532
M6_1	FirstPopTime	0.046005133	0.858373316
M6_2	FirstPopTime	0.046005133	0.858373316
M7_1	FirstPopTime	-0.105915711	0.358065566
M7_2	FirstPopTime	-0.025828587	0.890129645
M8_1	FirstPopTime	-0.620976143	0.660152352
M8_2	FirstPopTime	-0.620976143	0.660152352
M9_1	FirstPopTime	0.169150285	0.338323319
M9_2	FirstPopTime	0.126591024	0.40843085
M10_1	FirstPopTime	2.283112709	0.220460353
M10_2	FirstPopTime	2.283112709	0.220460353
M11_1	FirstPopTime	4.129058335	0.203230178
M12_1	FirstPopTime	0.052961663	0.10479017
M12_2	FirstPopTime	0.177607191	0.643744809
M13_1	FirstPopTime	3.613268987	0.039523661
M13_2	FirstPopTime	3.613268987	0.039523661
M14_1	FirstPopTime	0.013640447	0.939281345
M14_2	FirstPopTime	-0.064742791	0.77725428
M15_1	FirstPopTime	-0.652458507	0.330734818
M15_2	FirstPopTime	-0.652458507	0.330734818
M16_1	FirstPopTime	-0.024956486	0.918973685
M16_2	FirstPopTime	-0.001282704	0.995678809
M17_1	FirstPopTime	-0.105793797	0.791429265
M17_2	FirstPopTime	-0.333638329	0.479206271
M18_1	FirstPopTime	0.146404961	0.764535517
M18_2	FirstPopTime	0.138756992	0.78257714
M19_1	FirstPopTime	0.137025277	0.362019437
M19_2	FirstPopTime	0.137025277	0.362019437
M20_1	FirstPopTime	-0.044482335	0.566807914
M20_2	FirstPopTime	-1.314632181	0.251887664
M21_1	FirstPopTime	-0.017107116	0.896694376
M21_2	FirstPopTime	0.021605839	0.898534737
M22_1	FirstPopTime	-0.024354271	0.973780931
M22_2	FirstPopTime	-0.261058618	0.681486863
M23_1	FirstPopTime	-0.105040118	0.124553158
M23_2	FirstPopTime	-0.110375047	0.106245621
M24_1	FirstPopTime	0.140565882	0.952923866
M24_2	FirstPopTime	0.140565882	0.952923866
M25_1	FirstPopTime	-0.038562946	0.871906037
M25_2	FirstPopTime	0.051084471	0.492633921
M1_1	PoppedVolume	-17.33812857	0.319857373
M1_2	PoppedVolume	-20.88083054	0.03764273
M2_1	PoppedVolume	-0.024561123	0.992897891
M2_2	PoppedVolume	1.291339599	0.681382094
M3_1	PoppedVolume	15.08315611	0.013804102
M3_2	PoppedVolume	13.38597822	0.059525077
M4_2	PoppedVolume	-7.298746494	0.016845136
M5_1	PoppedVolume	13.02839919	0.058107234
M5_2	PoppedVolume	13.02839919	0.058107234
M6_1	PoppedVolume	0.718052623	0.805487671

M6_2	PoppedVolume	0.718052623	0.805487671
M7_1	PoppedVolume	-2.81706005	0.016845136
M7_2	PoppedVolume	-1.992095069	0.337029573
M8_1	PoppedVolume	-33.01708217	0.021531252
M8_2	PoppedVolume	-33.01708217	0.021531252
M9_1	PoppedVolume	2.628571194	0.180350525
M9_2	PoppedVolume	2.928631551	0.074808768
M10_1	PoppedVolume	2.395028278	0.912430083
M10_2	PoppedVolume	2.395028278	0.912430083
M11_1	PoppedVolume	17.17054707	0.650198661
M12_1	PoppedVolume	0.095740145	0.806302944
M12_2	PoppedVolume	-3.979371248	0.353132232
M13_1	PoppedVolume	20.14805522	0.344448971
M13_2	PoppedVolume	20.14805522	0.344448971
M14_1	PoppedVolume	3.333990474	0.078908905
M14_2	PoppedVolume	0.724002142	0.779846221
M15_1	PoppedVolume	5.32469773	0.487458944
M15_2	PoppedVolume	5.32469773	0.487458944
M16_1	PoppedVolume	1.958630224	0.475765501
M16_2	PoppedVolume	2.255512494	0.392579263
M17_1	PoppedVolume	5.912843115	0.17483789
M17_2	PoppedVolume	3.479425674	0.515094719
M18_1	PoppedVolume	-5.550569602	0.305596835
M18_2	PoppedVolume	-3.581809624	0.526136816
M19_1	PoppedVolume	-1.126869481	0.511409925
M19_2	PoppedVolume	-1.126869481	0.511409925
M20_1	PoppedVolume	0.6779876	0.437769904
M20_2	PoppedVolume	-0.510753784	0.969503058
M21_1	PoppedVolume	1.770803822	0.220068252
M21_2	PoppedVolume	2.181232545	0.241465189
M22_1	PoppedVolume	8.625701973	0.292069332
M22_2	PoppedVolume	0.255023898	0.971842062
M23_1	PoppedVolume	0.427093846	0.597682656
M23_2	PoppedVolume	0.313850814	0.700238734
M24_1	PoppedVolume	-26.44409516	0.315903643
M24_2	PoppedVolume	-26.44409516	0.315903643
M25_1	PoppedVolume	-0.907514353	0.736937644
M25_2	PoppedVolume	0.032384272	0.969654696
M1_1	Expandability	-1.532697886	0.337873453
M1_2	Expandability	-1.919031099	0.036916038
M2_1	Expandability	-0.009724299	0.969313906
M2_2	Expandability	0.088558941	0.758964012
M3_1	Expandability	1.357539879	0.016138986
M3_2	Expandability	1.203165388	0.065368928
M4_2	Expandability	-0.659314797	0.018925991
M5_1	Expandability	1.189903604	0.059025783
M5_2	Expandability	1.189903604	0.059025783
M6_1	Expandability	0.050238205	0.850913785
M6_2	Expandability	0.050238205	0.850913785
M7_1	Expandability	-0.254472378	0.018925991
M7_2	Expandability	-0.157470753	0.409927433
M8_1	Expandability	-2.999120475	0.022979687
M8_2	Expandability	-2.999120475	0.022979687
M9_1	Expandability	0.254606908	0.154630024
M9_2	Expandability	0.26203112	0.082940305
M10_1	Expandability	0.273813499	0.890828751
M10_2	Expandability	0.273813499	0.890828751
M11_1	Expandability	1.457845134	0.674487411
M12_1	Expandability	0.01065407	0.765688981
M12_2	Expandability	-0.389288375	0.320131592

M13_1	Expandability	1.97847404	0.309451114
M13_2	Expandability	1.97847404	0.309451114
M14_1	Expandability	0.307416623	0.076711246
M14_2	Expandability	0.044008424	0.85301079
M15_1	Expandability	0.492490013	0.483214099
M15_2	Expandability	0.492490013	0.483214099
M16_1	Expandability	0.166244302	0.509489383
M16_2	Expandability	0.19363893	0.423957778
M17_1	Expandability	0.551468712	0.166537743
M17_2	Expandability	0.338860459	0.488435269
M18_1	Expandability	-0.497545724	0.316637732
M18_2	Expandability	-0.354466069	0.49285579
M19_1	Expandability	-0.101324452	0.519417064
M19_2	Expandability	-0.101324452	0.519417064
M20_1	Expandability	0.056007136	0.485220815
M20_2	Expandability	-0.144003416	0.906314662
M21_1	Expandability	0.156984509	0.236376807
M21_2	Expandability	0.193557318	0.257483626
M22_1	Expandability	0.806656662	0.281655852
M22_2	Expandability	-0.010757982	0.987032843
M23_1	Expandability	0.038090505	0.607550058
M23_2	Expandability	0.02783288	0.709450033
M24_1	Expandability	-2.454247712	0.30935191
M24_2	Expandability	-2.454247712	0.30935191
M25_1	Expandability	-0.079204988	0.74902043
M25_2	Expandability	0.003086787	0.968428972
M1_1	UnpoppedNumber	-0.113727768	0.589308789
M1_2	UnpoppedNumber	-0.132693495	0.299990599
M2_1	UnpoppedNumber	0.008546492	0.794440132
M2_2	UnpoppedNumber	-0.010490646	0.7797764
M3_1	UnpoppedNumber	0.094832368	0.237683689
M3_2	UnpoppedNumber	0.086571047	0.332750302
M4_2	UnpoppedNumber	-0.042323973	0.289932197
M5_1	UnpoppedNumber	0.066608478	0.445181758
M5_2	UnpoppedNumber	0.066608478	0.445181758
M6_1	UnpoppedNumber	0.044694954	0.181459952
M6_2	UnpoppedNumber	0.044694954	0.181459952
M7_1	UnpoppedNumber	-0.016335568	0.289932197
M7_2	UnpoppedNumber	0.014611971	0.559156612
M8_1	UnpoppedNumber	-0.180054636	0.336381055
M8_2	UnpoppedNumber	-0.180054636	0.336381055
M9_1	UnpoppedNumber	-0.008343537	0.730470488
M9_2	UnpoppedNumber	-0.004315374	0.836597087
M10_1	UnpoppedNumber	-0.148508599	0.564077736
M10_2	UnpoppedNumber	-0.148508599	0.564077736
M11_1	UnpoppedNumber	0.163605605	0.717091409
M12_1	UnpoppedNumber	0.00528111	0.242557938
M12_2	UnpoppedNumber	0.059580544	0.23752925
M13_1	UnpoppedNumber	0.45287871	0.058925081
M13_2	UnpoppedNumber	0.45287871	0.058925081
M14_1	UnpoppedNumber	0.014226936	0.552428656
M14_2	UnpoppedNumber	-0.01087847	0.723990746
M15_1	UnpoppedNumber	-0.016811717	0.855308456
M15_2	UnpoppedNumber	-0.016811717	0.855308456
M16_1	UnpoppedNumber	0.010612975	0.747665985
M16_2	UnpoppedNumber	0.012491498	0.694501231
M17_1	UnpoppedNumber	0.094115068	0.060161735
M17_2	UnpoppedNumber	0.104867219	0.081484086
M18_1	UnpoppedNumber	0.014791514	0.82244423
M18_2	UnpoppedNumber	-0.011406649	0.8664333

M19_1	UnpoppedNumber	-0.001844147	0.928737759
M19_2	UnpoppedNumber	-0.001844147	0.928737759
M20_1	UnpoppedNumber	0.004410178	0.674443173
M20_2	UnpoppedNumber	-0.028164421	0.859356064
M21_1	UnpoppedNumber	-0.004754785	0.788581392
M21_2	UnpoppedNumber	-0.012765077	0.57356909
M22_1	UnpoppedNumber	-0.042663309	0.667911064
M22_2	UnpoppedNumber	-0.115605647	0.160871711
M23_1	UnpoppedNumber	-8.75E-04	0.928036082
M23_2	UnpoppedNumber	-0.00161503	0.868182137
M24_1	UnpoppedNumber	-0.091303796	0.775584304
M24_2	UnpoppedNumber	-0.091303796	0.775584304
M25_1	UnpoppedNumber	0.050054266	0.099955841
M25_2	UnpoppedNumber	9.28E-04	0.927053441
M1_1	UnpoppedWeight	-0.020236491	0.482197667
M1_2	UnpoppedWeight	-0.020000475	0.252770738
M2_1	UnpoppedWeight	8.31E-04	0.853736386
M2_2	UnpoppedWeight	-0.001855948	0.718302648
M3_1	UnpoppedWeight	0.009944226	0.373303449
M3_2	UnpoppedWeight	0.008492116	0.493288531
M4_2	UnpoppedWeight	-0.005305057	0.335961331
M5_1	UnpoppedWeight	0.009009338	0.452109271
M5_2	UnpoppedWeight	0.009009338	0.452109271
M6_1	UnpoppedWeight	0.006054335	0.187822334
M6_2	UnpoppedWeight	0.006054335	0.187822334
M7_1	UnpoppedWeight	-0.002047566	0.335961331
M7_2	UnpoppedWeight	0.002012758	0.557820365
M8_1	UnpoppedWeight	-0.023411147	0.363597332
M8_2	UnpoppedWeight	-0.023411147	0.363597332
M9_1	UnpoppedWeight	-0.001108157	0.738964146
M9_2	UnpoppedWeight	-5.42E-04	0.850462367
M10_1	UnpoppedWeight	-0.021101923	0.55030939
M10_2	UnpoppedWeight	-0.021101923	0.55030939
M11_1	UnpoppedWeight	0.015777136	0.799377745
M12_1	UnpoppedWeight	6.86E-04	0.270556775
M12_2	UnpoppedWeight	0.007732409	0.265770432
M13_1	UnpoppedWeight	0.042442543	0.216790125
M13_2	UnpoppedWeight	0.042442543	0.216790125
M14_1	UnpoppedWeight	7.16E-04	0.828632304
M14_2	UnpoppedWeight	3.53E-04	0.933691349
M15_1	UnpoppedWeight	-0.001198473	0.924603289
M15_2	UnpoppedWeight	-0.001198473	0.924603289
M16_1	UnpoppedWeight	0.001662315	0.713395655
M16_2	UnpoppedWeight	0.001867233	0.668719649
M17_1	UnpoppedWeight	0.01174637	0.092153953
M17_2	UnpoppedWeight	0.013073146	0.118203521
M18_1	UnpoppedWeight	-7.50E-04	0.934053845
M18_2	UnpoppedWeight	-0.004320451	0.641255627
M19_1	UnpoppedWeight	-7.47E-05	0.978960271
M19_2	UnpoppedWeight	-7.47E-05	0.978960271
M20_1	UnpoppedWeight	1.42E-04	0.921938205
M20_2	UnpoppedWeight	8.63E-04	0.968490173
M21_1	UnpoppedWeight	-2.71E-04	0.911612855
M21_2	UnpoppedWeight	-0.001319669	0.672636383
M22_1	UnpoppedWeight	-0.007672194	0.573023706
M22_2	UnpoppedWeight	-0.016706543	0.137997375
M23_1	UnpoppedWeight	-3.04E-04	0.81904453
M23_2	UnpoppedWeight	-3.99E-04	0.764965115
M24_1	UnpoppedWeight	-0.022019474	0.615201466
M24_2	UnpoppedWeight	-0.022019474	0.615201466

M25_1	UnpoppedWeight	0.006085882	0.150609848
M25_2	UnpoppedWeight	-2.30E-05	0.986830267
M1_1	FlakeCircularity	-0.023890395	0.233396493
M1_2	FlakeCircularity	0.005469273	0.667385944
M2_1	FlakeCircularity	-0.004330386	0.158208119
M2_2	FlakeCircularity	-0.005493698	0.113701794
M3_1	FlakeCircularity	-8.07E-04	0.920334475
M3_2	FlakeCircularity	-0.004644341	0.599378752
M4_2	FlakeCircularity	-0.00542243	0.156464008
M5_1	FlakeCircularity	0.002505891	0.770758366
M5_2	FlakeCircularity	0.002505891	0.770758366
M6_1	FlakeCircularity	0.004376134	0.17952826
M6_2	FlakeCircularity	0.004376134	0.17952826
M7_1	FlakeCircularity	-0.002092868	0.156464008
M7_2	FlakeCircularity	0.001166244	0.633428224
M8_1	FlakeCircularity	-0.015378906	0.402011411
M8_2	FlakeCircularity	-0.015378906	0.402011411
M9_1	FlakeCircularity	-0.003200392	0.158279851
M9_2	FlakeCircularity	-0.003311382	0.083896512
M10_1	FlakeCircularity	0.00999463	0.691683091
M10_2	FlakeCircularity	0.00999463	0.691683091
M11_1	FlakeCircularity	0.064134787	0.126714406
M12_1	FlakeCircularity	-4.28E-04	0.335866893
M12_2	FlakeCircularity	0.004631014	0.351910762
M13_1	FlakeCircularity	-0.005697652	0.821110288
M13_2	FlakeCircularity	-0.005697652	0.821110288
M14_1	FlakeCircularity	9.78E-04	0.676615257
M14_2	FlakeCircularity	0.001499982	0.616569963
M15_1	FlakeCircularity	-0.01717468	0.036194012
M15_2	FlakeCircularity	-0.01717468	0.036194012
M16_1	FlakeCircularity	0.002143282	0.501997787
M16_2	FlakeCircularity	0.002401834	0.434098011
M17_1	FlakeCircularity	0.0031539	0.545896007
M17_2	FlakeCircularity	8.31E-04	0.894380742
M18_1	FlakeCircularity	0.013011804	0.024484632
M18_2	FlakeCircularity	0.01026207	0.100342863
M19_1	FlakeCircularity	0.002794171	0.145918392
M19_2	FlakeCircularity	0.002794171	0.145918392
M20_1	FlakeCircularity	-0.001007965	0.316147086
M20_2	FlakeCircularity	-0.025908004	0.073693927
M21_1	FlakeCircularity	0.001654344	0.32944064
M21_2	FlakeCircularity	0.001706228	0.437589917
M22_1	FlakeCircularity	-0.008770882	0.358876875
M22_2	FlakeCircularity	-0.009291797	0.254542953
M23_1	FlakeCircularity	-9.60E-04	0.298650732
M23_2	FlakeCircularity	-0.00104427	0.258154821
M24_1	FlakeCircularity	-0.05195097	0.075687921
M24_2	FlakeCircularity	-0.05195097	0.075687921
M25_1	FlakeCircularity	-0.003044023	0.322692026
M25_2	FlakeCircularity	-6.04E-04	0.53772538
M1_1	PericarpRetention	-0.152263953	0.539451238
M1_2	PericarpRetention	0.022779889	0.88282854
M2_1	PericarpRetention	-0.006774268	0.861178019
M2_2	PericarpRetention	0.034896038	0.424958099
M3_1	PericarpRetention	0.069262544	0.473298843
M3_2	PericarpRetention	0.034942799	0.744890301
M4_2	PericarpRetention	-0.072770791	0.112112161
M5_1	PericarpRetention	0.156534562	0.113090367
M5_2	PericarpRetention	0.156534562	0.113090367
M6_1	PericarpRetention	-0.027915804	0.491697699

M6_2	PericarpRetention	-0.027915804	0.491697699
M7_1	PericarpRetention	-0.028086972	0.112112161
M7_2	PericarpRetention	0.006324068	0.83143945
M8_1	PericarpRetention	-0.363169817	0.086309146
M8_2	PericarpRetention	-0.363169817	0.086309146
M9_1	PericarpRetention	0.024125077	0.392282346
M9_2	PericarpRetention	0.025562404	0.289185229
M10_1	PericarpRetention	0.295208213	0.324534541
M10_2	PericarpRetention	0.295208213	0.324534541
M11_1	PericarpRetention	-0.019594033	0.970746428
M12_1	PericarpRetention	-0.003040252	0.577334069
M12_2	PericarpRetention	0.057282291	0.340924116
M13_1	PericarpRetention	-0.012276759	0.967919174
M13_2	PericarpRetention	-0.012276759	0.967919174
M14_1	PericarpRetention	0.049270552	0.062440856
M14_2	PericarpRetention	0.021787629	0.546848044
M15_1	PericarpRetention	-0.044413917	0.682207588
M15_2	PericarpRetention	-0.044413917	0.682207588
M16_1	PericarpRetention	0.03697286	0.333484483
M16_2	PericarpRetention	0.038423728	0.296102103
M17_1	PericarpRetention	0.005645941	0.929363238
M17_2	PericarpRetention	-0.040769182	0.588254812
M18_1	PericarpRetention	-0.014816746	0.848966845
M18_2	PericarpRetention	-0.037470591	0.638214494
M19_1	PericarpRetention	-0.020684917	0.387660383
M19_2	PericarpRetention	-0.020684917	0.387660383
M20_1	PericarpRetention	0.02310344	0.042688996
M20_2	PericarpRetention	-0.069676666	0.709620282
M21_1	PericarpRetention	0.018650953	0.364925044
M21_2	PericarpRetention	0.024281863	0.35879935
M22_1	PericarpRetention	0.150936155	0.183175085
M22_2	PericarpRetention	0.068192901	0.497377531
M23_1	PericarpRetention	-0.002356901	0.836605059
M23_2	PericarpRetention	-0.00335189	0.770072057
M24_1	PericarpRetention	-0.181697391	0.629546655
M24_2	PericarpRetention	-0.181697391	0.629546655
M25_1	PericarpRetention	-0.02600128	0.489864422
M25_2	PericarpRetention	-2.28E-04	0.984820025
