# Supplementary figures and images for: SSR-Based Genetic Diversity, Population Structure, and Marker–Trait Associations for Popping-Related Traits in Popcorn Germplasm
Source: Genes (Basel). 2026 Jun 12;17(6):690. doi: 10.3390/genes17060690 (PMC13300098; doi:10.3390/genes17060690)

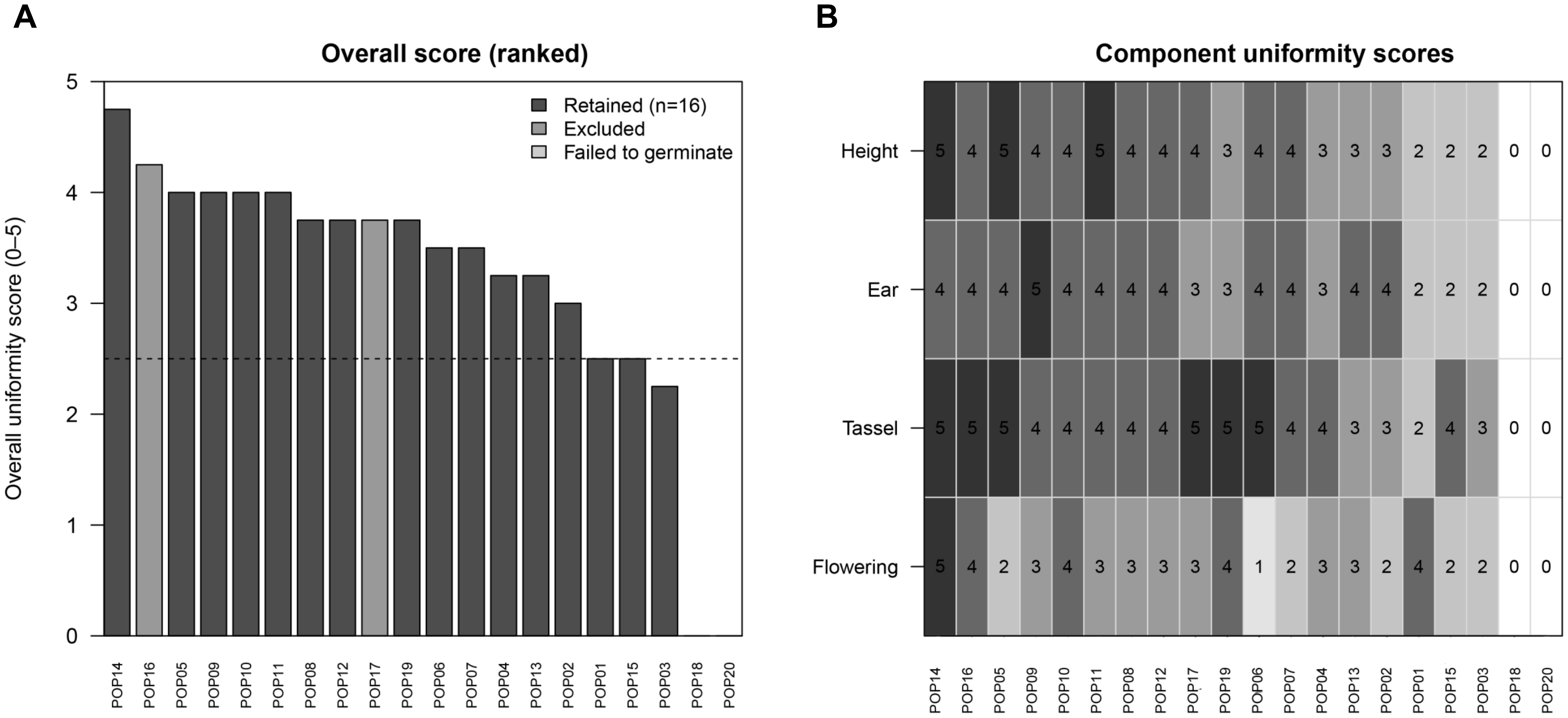

Supplement: Supplementary file 1 [file genes-17-00690-s001.zip › Figure_S1.png]
